# The karyosphere capsule in *Rana temporaria* oocytes contains structural and DNA-binding proteins

**DOI:** 10.1080/19491034.2018.1530935

**Published:** 2018-10-01

**Authors:** Nadya Ilicheva, Olga Podgornaya, Dmitry Bogolyubov, Galina Pochukalina

**Affiliations:** a Laboratory of Cell Morphology, Institute of Cytology of the Russian Academy of Sciences, St. Petersburg, Russia; b Department of Cytology and Histology, Faculty of Biology, Saint Petersburg State University, St. Petersburg, Russia; c Laboratory of Biomedical Cell Technology, School of Biomedicine, Far Eastern Federal University, Vladivostok, Russia

**Keywords:** Karyosphere, karyosphere capsule, oogenesis, *Rana temporaria*, transcription, nuclear actin, lamins, nucleoporins, ATRX, topoisomerase II

## Abstract

During the last stages of oogenesis, oocyte chromosomes condense and come close together, forming the so-called karyosphere. Karyosphere formation is accompanied by an essential decrease in transcriptional activity. In the grass frog *Rana temporaria*, the karyosphere is surrounded by an extrachromosomal capsule that separates the chromosomes from the rest of the nucleoplasm. The karyosphere capsule (KC) of *R. temporaria* has been investigated in detail at the ultrastructural level, but its protein composition remained largely unknown. We demonstrate here that nuclear actin, especially F-actin, as well as lamins A/C and B are the most abundant proteins of the KC. Key proteins of nuclear pore complexes, such as Nup93 and Nup35, are also detectable in the KC. New antibodies recognizing the telomere-binding protein TRF2 allowed us to localize TRF2 in nuclear speckles. We also found that the *R. temporaria* KC contains some proteins involved in chromatin remodeling, including topoisomerase II and ATRX. Thus, we believe that KC isolates the chromosomes from the rest of the nucleoplasm during the final period of oocyte growth (late diplotene) and represents a specialized oocyte nuclear compartment to store a variety of factors involved in nuclear metabolism that can be used in future early development.

**Abbreviations:** BrUTP: 5-bromouridine 5’-triphosphate; CytD: cytochalasin D; IGCs: interchromatin granule clasters; IgG: immunoglobulin G; KC: karyosphere capsule; Mw: molecular weight; NE: nuclear envelope; PBS: phosphate buffered saline; SDS-PAGE: sodium dodecyl sulfate polyacrylamide gel electrophoresis; Topo II: topoisomerase II

## Introduction

During oogenesis in many but not all animals, both invertebrates and vertebrates, oocyte chromosomes condense and assemble together in a more or less compact structure, known as a karyosphere, which occupies a limited area of the large oocyte nucleus at the diplotene stage of meiotic prophase [1,2]. The karyosphere structure is species-specific and varies from a simple compact ‘knot’ of chromosomes to complex superstructural formations that include various extrachromosomal elements. In general, three types of the karyosphere can be distinguished: (i) the karyosphere with an external extrachromosomal capsule, (ii) the karyosphere without a capsule, also known as a karyosome, and (iii) the inverted karyosphere, when chromosomes are attached externally to an extrachromosomal central body. Central bodies are highly variable in different organisms, such as birds or mammals (reviewed in [2]).

Large-scale chromatin remodeling in the oocyte nucleus during meiotic arrest at the diplotene stage accompanied by karyosphere formation is necessary for further meiotic events to occur properly [3,4]. In particular, the close arrangement of chromosomes with respect to each other is essential for correct formation of the spindle apparatus [5]. The karyosphere develops after a prolonged period of active RNA synthesis, and the transcriptional activity of oocyte chromosomes decreases significantly during karyosphere formation (reviewed in [1,2]). Histone modifications are implicated in chromatin remodeling during karyosphere formation, providing an epigenetic mechanism for the developmental control of global gene expression during oogenesis [6,7].

In the grass frog, *Rana temporaria*, a highly complicated multilayer structure called the karyosphere capsule (KC) is formed around the condensed chromosomes and separates them from the rest of the nucleoplasm in late oocytes. This leads to the appearance of something similar to ‘a nucleus within the nucleus’. The *R. temporaria* KC with encapsulated chromosomes can be isolated from the oocyte nucleus as a single entity, which indicates that all components of the KC are bound together rather firmly [8].

The KC is always composed of a filamentous material; other structures, such as different nuclear bodies, nucleoli and derivatives of synaptonemal complexes, can be found in association with the KC [1]. The KC is an obligatory attribute of oocytes in a particular species in which the KC develops, but it may be absent in another closely related species from the same family. The KC is traditionally considered as a specialized part of the nucleoskeleton required for additional support to the karyosphere in the enormous oocyte nucleus [1], but the true biological significance of the KC is unknown.

The dynamics of KC formation in *R. temporaria* oocytes was described in detail ~ 100 years ago by Wagner [9]; the term ‘Wagner’s capsule’ was occasionally used for this structure [10]. The grass frog demonstrates a discernable seasonality of breeding. The KC begins to form around the lampbrush chromosomes that gradually lose their lateral loops during the autumn-winter period, namely in vitellogenic oocytes of stage 5 according to the nomenclature proposed by Duryee [11]. The *R. temporaria* KC reaches its maximum complexity and width of 150–200 μm in March, at stage 6 oocytes just before ovulation, when the compact ‘knot’ of chromosomes is entirely enclosed within the KC.

The KC structure in *R. temporaria* oocytes has been described previously using light and electron microscopy [10,12–14]. A portion of the capsule is formed by a filamentous material, which interweaves the chromosomes and protrudes into a zone occupied by numerous amplified nucleoli. The nucleoli as well as different ribonucleoprotein granules and nuclear bodies, some possessing a meshwork of 25-nm granules resembling interchromatin granules (IGCs, SC35 domains, speckles), have been observed in association with the fully developed KC.

A striking feature of the middle part of the *R. temporaria* KC is the presence of peculiar electron-dense fibrous strands, 40–50 nm in thickness, called ‘pseudomembranes’ [1,12]. Each ‘pseudomembrane’ represents a row of annulated structures linked not by membranes but embedded in a finely fibrillar matrix. At the ultrastructural level, these annuli are exactly similar to the pore complexes of the nuclear envelope (NE) [12]. In another frog from the same family Ranidae, the marsh frog *Pelophylax ridibundus*, formerly known as *R. ridibunda*, the autonomous pore complexes accumulate inside the mass of condensed chromosomes, forming a central body of the inverted karyosphere. No filamentous KC appears in this case [15]. Beyond amphibians, annulated ‘pseudomembranes’ have also been described in the KC of mosquitos [16], but not the red flour beetle *Tribolium castaneum* [17]. In *R. temporaria*, the autonomous pore complexes are associated with fine fibrils, 7–8 nm in thickness, which presumably originate from the central elements of synaptonemal complexes [12]. For comparison, the KC of mosquito oocytes contains multilayered ‘polycomplexes’, which represent multiple stacks of abnormal synaptonemal complexes [18]. The protein components of *R. temporaria* KC have not yet been identified. Thus, the karyosphere and its capsule have to be re-examined using modern cytological and cytochemical methods.

Late *R. temporaria* diplotene oocytes at stages 5 and 6 with the chromosomes packed into a capsule-surrounded karyosphere in the middle of the nucleus provide the possibility of isolating NE manually and checking the telomere-binding activity in NE extracts by gel mobility shift assay with telomeric DNA. As a result, previous studies have identified a membrane telomere-binding protein [19,20], found to be the telomeric repeat factor TRF2 [21–24]. A polyclonal antibody raised against TRF2-telomeric DNA complexes (TRF2-DNA) recognized a single protein of about 70 kDa (TRF2) in the NE and among the proteins of liver cell nuclei, while an antibody against telobox peptide (Myb/SANT domain) of TRF2 recognized the same protein of 70 kDa (TRF2) among NE proteins as well as a 60 kDa protein (TRF1) in the inner part of the oocyte nucleus. Thus, the specificity of these antibodies is not sufficient to distinguish different TRFs. Both antibodies also label some minor zones at ~ 100 kDa [25]. At the microscopic level (immunofluorescence and immunogold electron microscopy) the TRF2-DNA antibody stains the NE but not the inner part of the oocyte nucleus [20,25].

TRF2 has a specific poorly characterized linker region (udTRF2) between its homodimerization and DNA-binding domains, which differs it from TRF1. Some lines of evidence have shown that this region could be involved in TRF2 interactions with the nuclear lamina [25] The recombinant protein udTRF2 and an antibody against it (anti-udTRF2) have been generated and characterized [26,27]. This antibody happens to be more specific for the unprocessed high molecular weight (Mw) form of TRF2 and was used in the current work.

In this study, we used two very late stages of *R. temporaria* oocyte development (Duryee’s stages 5 and 6) when the KC is fully developed in the nucleus. The aim of the present work was to answer the following questions: (i) Is there a difference in actin forms and location in the late oocyte nucleus? (ii) What is the position of some DNA-binding proteins in the karyosphere? (iii) Do the structures visible as a membrane remnant contain the ordinary components of nuclear envelope? (iv) What is the position of the telomere-binding factor 2 (TRF2) in the late oocyte nucleus?

## Materials and methods

### Oocyte nuclei isolation

Vitellogenic oocytes (5 or 6 stage according to Duryee [11]) were obtained from *R. temporaria* mature females (three years old) and kept in OR2 medium (82.5 mM NaCl, 2.5 mM KCl, 1 mM CaCl_2_, 1 mM MgCl_2_, 1 mM Na_2_HPO_4_, 5 mM HEPES, pH 7.6). All animal procedures were performed according to the rules of biomedical ethics (protocol 264, 5 March 2011) certified by the Russian Academy of Sciences Committee on Bioethics. Nuclei were isolated manually according to a previously described method [28]. Briefly, oocytes were placed in a Petri dish with a 5:1+PO_4_ solution (83 mM KCl, 17 mM NaCl, 6.5 mM Na_2_HPO_4_, 3.5 mM KH_2_PO_4_), and the nuclei were isolated from oocytes under a binocular microscope using a titan needle and forceps. The nuclei were washed with 5:1+PO_4_ solution and processed for immunofluorescent staining.

### Immunofluorescent staining

Nuclei were fixed in 4% paraformaldehyde on PBS with 0.2% Triton X-100 for 20–40 min at room temperature and washed with PBS three times. To avoid unspecific binding, the samples were incubated in 10% bovine fetal serum for 10 min. Nuclei samples were incubated with primary antibodies at 4^о^C in a moist chamber overnight. The primary antibodies are listed in the Table 1. Preparations were washed with PBS 4 × 10 min and incubated with secondary antibodies for 1.5 h at room temperature. After washing with PBS, the samples were mounted in Vectashield medium with DAPI (1:1000) to visualize DNA-containing structures and examined under a Leica TCS SP5 confocal laser scanning microscope equipped with an argon (488 nm) and helium-neon (543, 633 nm) lasers and a 40× objective (aperture 1.25).

**Table 1.** Primary antibodies.

Antibody	Antigen	Source
Goat polyclonal anti-lamin В (Sc6217)	C-end of lamin B	Santa Cruz, US
Mouse monoclonal anti-lamin А (Ab8980)	Amino-acid residues 598–611 of lamin A	Abcam, UK
Rabbit polyclonal anti-lamin С (Ab8981)	Last 8 amino-acid residues on the C-end of lamin C	Abcam, UK
Mouse monoclonal anti-actin (MAB1501R, clone C4)	Amino acid residues 50–70 of actin	Millipore, US
Rabbit polyclonal anti-actin (A2103)	First nine amino acid residues of the N-terminal region of actin	Sigma, US
Rabbit polyclonal anti-actin (A2066)	Last 11 amino acid residues at the C-end of actin	Sigma, US
Mouse monoclonal anti-ATRX (sc-15408)	Amino acid residues 2193–2492 mapping at the C-terminus	Santa Cruz, US
Rabbit polyclonal anti-TopoII (AV04007)	C-end of topoisomerase II	Sigma, US
Rabbit polyclonal anti-TRF2 (ab4182)	Amino acid residues 250 – 350 of TRF2	Abcam, UK
Guinea pig polyclonal Anti-udTRF2	Amino acid residues 245–445 of TRF2	[26,27]
Mouse monoclonal anti-Nup93 (sc-374399)	Amino acid residues 1–300 of Nup93	Santa Cruz, US
Goat polyclonal anti-Nup35 (Sc-74762)	A peptide mapping within an internal region of Nup35	Santa Cruz, US
Rabbit polyclonal anti-H3K9me3 (Ab8898)	Synthetic peptide within Human Histone H3 amino acid residues 1–100 (N terminal) (tri-methyl K9)	Abcam, UK
Mouse monoclonal anti-SC35 (S4045)	Phospho-epitope of SC-35	Sigma, US
Mouse monoclonal anti-BrdU (clone BU-33)	Bromodeoxyuridine conjugated to KLH	Sigma, US

### BrUTP microinjections

The oocytes were placed in a Petri dish with OR2 media. 18,4 nl of 100 mM BrUTP (Sigma) in a transcription buffer (140 mM KCl, 2 mM PIPES, pH 7.4) was injected into the oocytes using microinjector (Nanoject II, Drummond). Oocytes were incubated in OR2 media for 1 hour at room temperature (22°C). After incubation, the nuclei were isolated as described earlier, washed with 5:1+PO_4_ solution and processed for immunofluorescent staining. The primary antibody was mouse monoclonal antibody against BrdU (clone BU-33, Sigma) at a dilution of 1:600. As a control, a portion of the nuclei was treated with 20 μg/ml RNAse A for 1 hour at room temperature before immunostaining.

### TRITC-phalloidin staining

The nuclei were isolated from oocytes, then washed and fixed as described above. After fixation, the nuclei were washed with PBS 3 × 10 min and incubated in TRITC-phalloidin solution (10 μg/ml) for 1 h in a moist chamber at room temperature. After staining, the nuclei were washed with PBS 4 × 10 min and mounted in Vectashield medium with DAPI.

### Cytochalasin D treatment

Oocytes were incubated in CytD-containing OR2 medium for 2.5 h at room temperature. CytD (Sigma) was diluted to a final concentration of 4 μM. After incubation, the nuclei were isolated from the oocytes and fixed in 4% paraformaldehyde in PBS containing 0.2% Triton X-100. The fixed nuclei were either mounted in Vectashield with DAPI or stained with antibodies to actin (A2103, dilution 1:50, and A2066, dilution 1:50) as described above.

### Electrophoresis and immunoblotting

The nuclei were washed with 5:1+PO_4_ solution twice and incubated in 5:1+PO_4_ solution containing 1 mM CaCl_2_ for 10 min. This treatment induced shrinkage of the nucleoplasm and allowed for easier separation of the nuclear envelope (NE) and nuclear contents (gel). All solutions contained 1 mM PMSF. The samples of NE and gels were dissolved in Laemmli sample buffer and used for SDS-PAGE and western blot. The primary antibodies (anti-TRF2 from Abcam and anti-udTRF2) were diluted 1:500, and secondary antibodies, i.e. goat anti-guinea pig or goat anti-rabbit IgG (whole molecule) conjugated to alkaline phosphatase (Sigma) were diluted 1:10,000. Protein isolation and antibody production protocols have been published [26,27].

## Results

**Figure 1.**
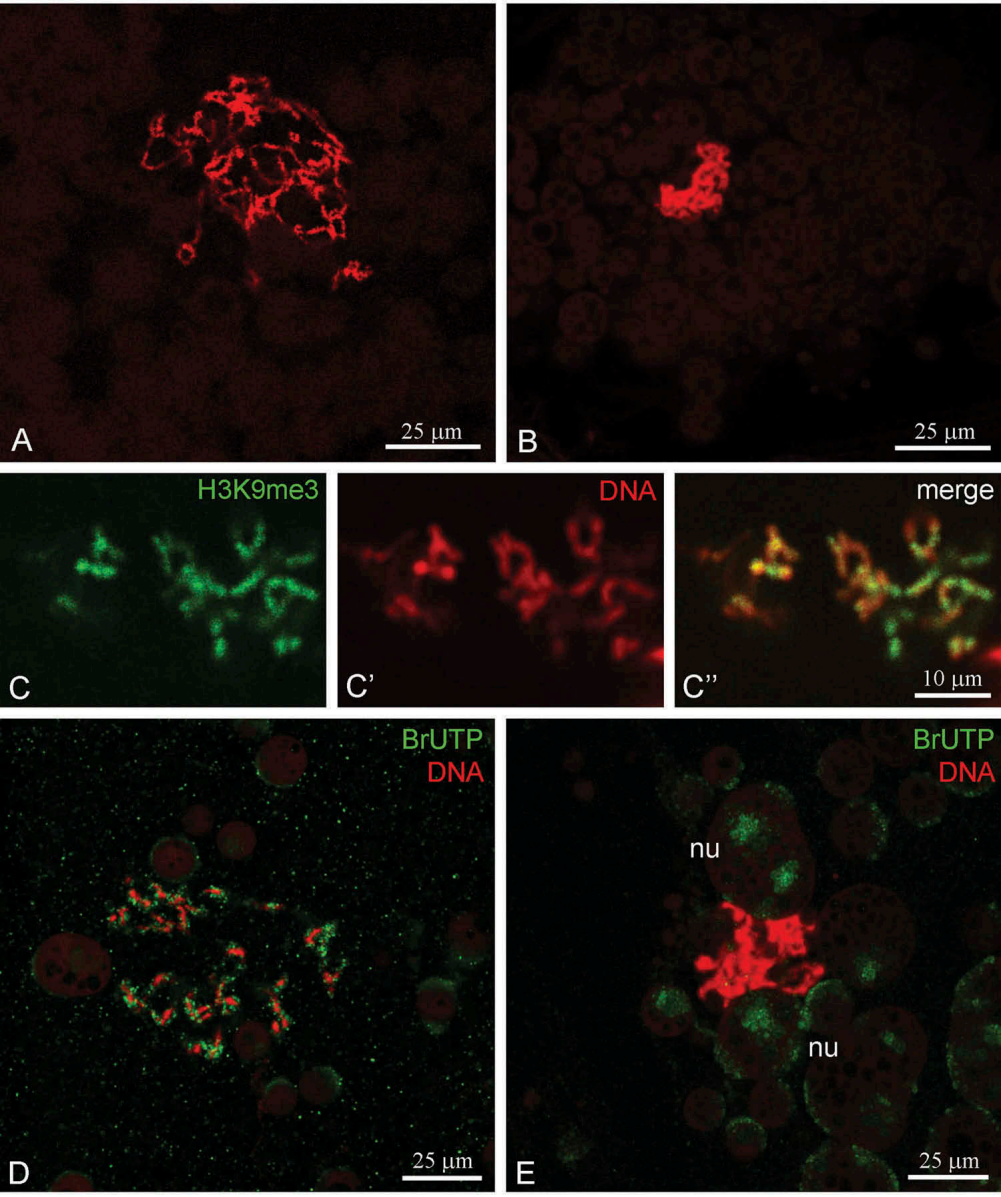
Karyosphere in *Rana temporaria* late oocytes. A, B, Morphology of the karyosphere in stage 5 oocyte (a) and stage 6 oocyte (b). DAPI staining, artificial red color. C-C’’, Localization of modified histone H3K9me3 (*green*) in the karyosphere of stage 5 oocyte, DNA is counterstained with DAPI (*red*). D, E, Incorporation of BrUTP (*green*) in the karyosphere and nucleoli of stage 5 oocyte (d) and stage 6 oocyte (e), DNA is counterstained with DAPI (*red*); nu, nucleoli.

At stage 4, according to the nomenclature proposed for frog oogenesis [11], the nucleus of *R. temporaria* vitellogenic oocytes contains a fully assembled karyosphere that represents a loose ‘knot’ of thin threadlike chromosomes assembled together in the nucleus. Further development of the karyosphere at stages 5–6 is accompanied by greater compaction of chromosomes within the karyosphere (Figure 1(a,b)). The karyosphere reaches maximum compactness in stage 6 oocytes that are ready to ovulate. At all the stages studied, oocyte chromosomes assembled into a karyosphere demonstrate a high level of histone H3 tri-methylated at lysine 9 (H3K9me3), and H3K9me3 staining coincided perfectly with DAPI staining (Figure 1(c)), indicating a rather inactive DNA state. However, the maximum cessation of karyosphere transcription activity is reached only at the end of oocyte development (stage 6). Microinjection experiments with BrUTP revealed residual but still clearly detectable transcriptional activity in the chromosomes assembled into the karyosphere in stage 5 oocytes (Figure 1(d)), but the karyosphere did not incorporate BrUTP in later (stage 6) oocytes (Figure 1(e)). Residual transcription was observed only in amplified nucleoli in stage 6 oocytes.

**Figure 2.**
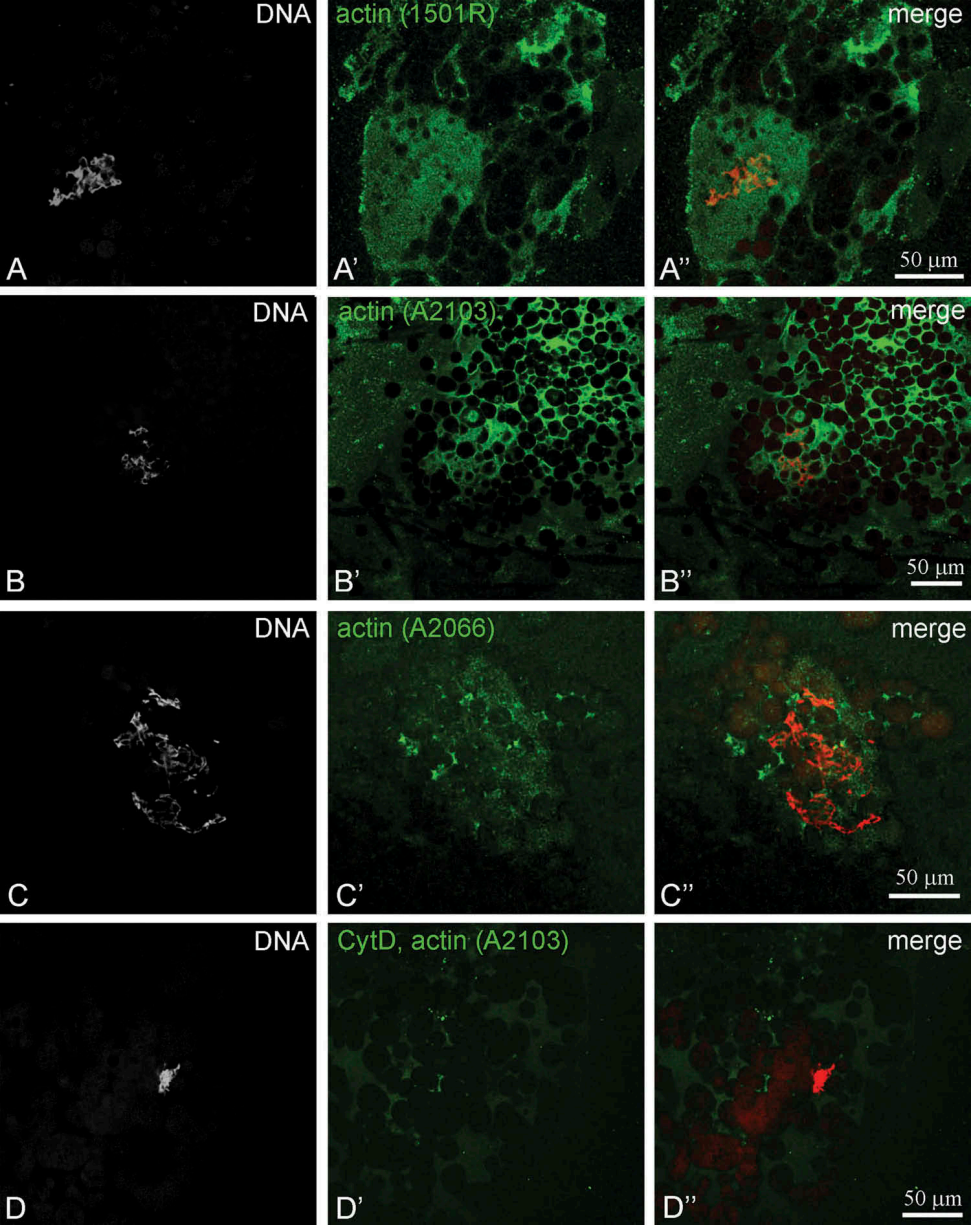
Localization of actin in the karyosphere capsule of *Rana temporaria* oocytes with antibodies against different epitopes of actin molecules. (a) Stage 6 oocyte nucleus stained with Mab1501R antibody against amino acid residues 50–70; (b) Stage 5 oocyte nucleus stained with A2103 antibody against the N-terminus; (c) Stage 5 oocyte nucleus stained with A2066 antibody against the C-terminus. (d) The karyosphere with capsule after cytochalasin D (CytD) treatment stained with A2103 antibody. DNA is counterstained with DAPI.

Immunocytochemical staining with anti-actin antibodies showed that actin represents one of the main constituents of the *R. temporaria* KC (Figure 2). Three antibodies against different epitopes of actin were used to trace the different forms of actin that can exist within the oocyte nucleus. Both at 5 and 6 stages of oocyte development, two anti-actin antibodies, one against a fragment situated at some distance from the N-terminus and the other against the first nine amino acid residues at the N-terminus, both exhibited similar results: the protein was distributed throughout the KC with some local agglomerates (Figure 2(a,b)). A difference in the actin distribution was observed with the antibody against the C-terminus at stage 5. The staining did not include the entire fibrous component of KC and was exhibited as weak staining. Several small bright dots were also visible in the vicinity of the chromosomes (Figure 2(c)). No perceptible differences in actin staining were observed at stage 6; all three antibodies demonstrated rather uniform staining of the KC.

**Figure 3.**
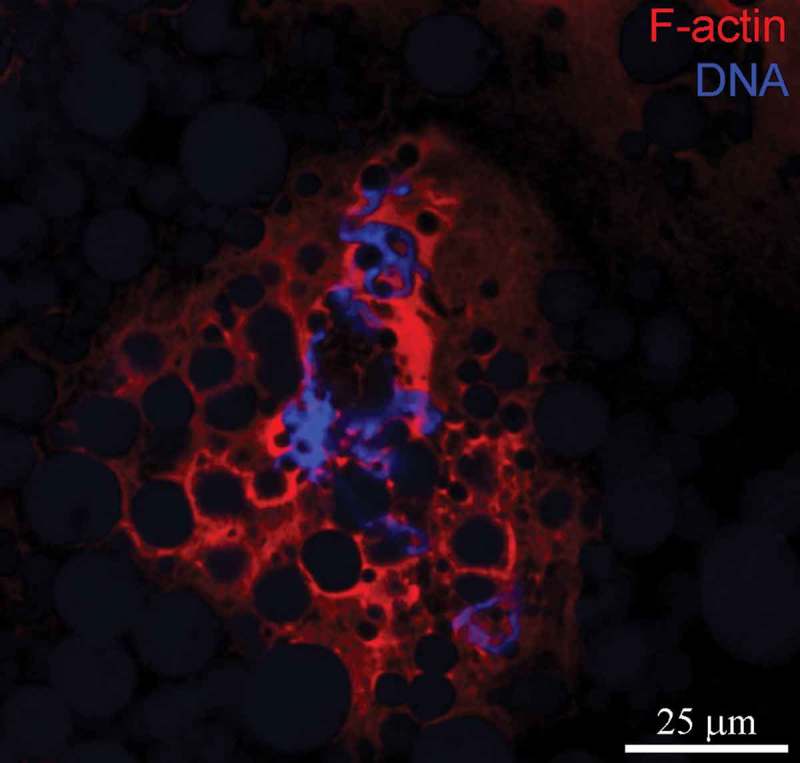
*Rana temporaria* karyosphere with capsule after TRITC-phalloidin staining for F-actin (*red*). DNA (karyosphere) is counterstained with DAPI (*blue*).

In contrast, a significant difference in actin staining was observed after treatment of stage 5 oocytes with cytochalasin D (CytD), suggesting that a large portion of actin in the KC is in the polymeric form (Figure 2(d)). Antibodies against the N-terminus revealed the destruction of actin filaments and the compaction of chromosomes. The antibody against the C-terminus did not produce any staining. Staining with TRITC-phalloidin confirmed the presence of a large amount of F-actin in the KC (Figure 3).

**Figure 4.**
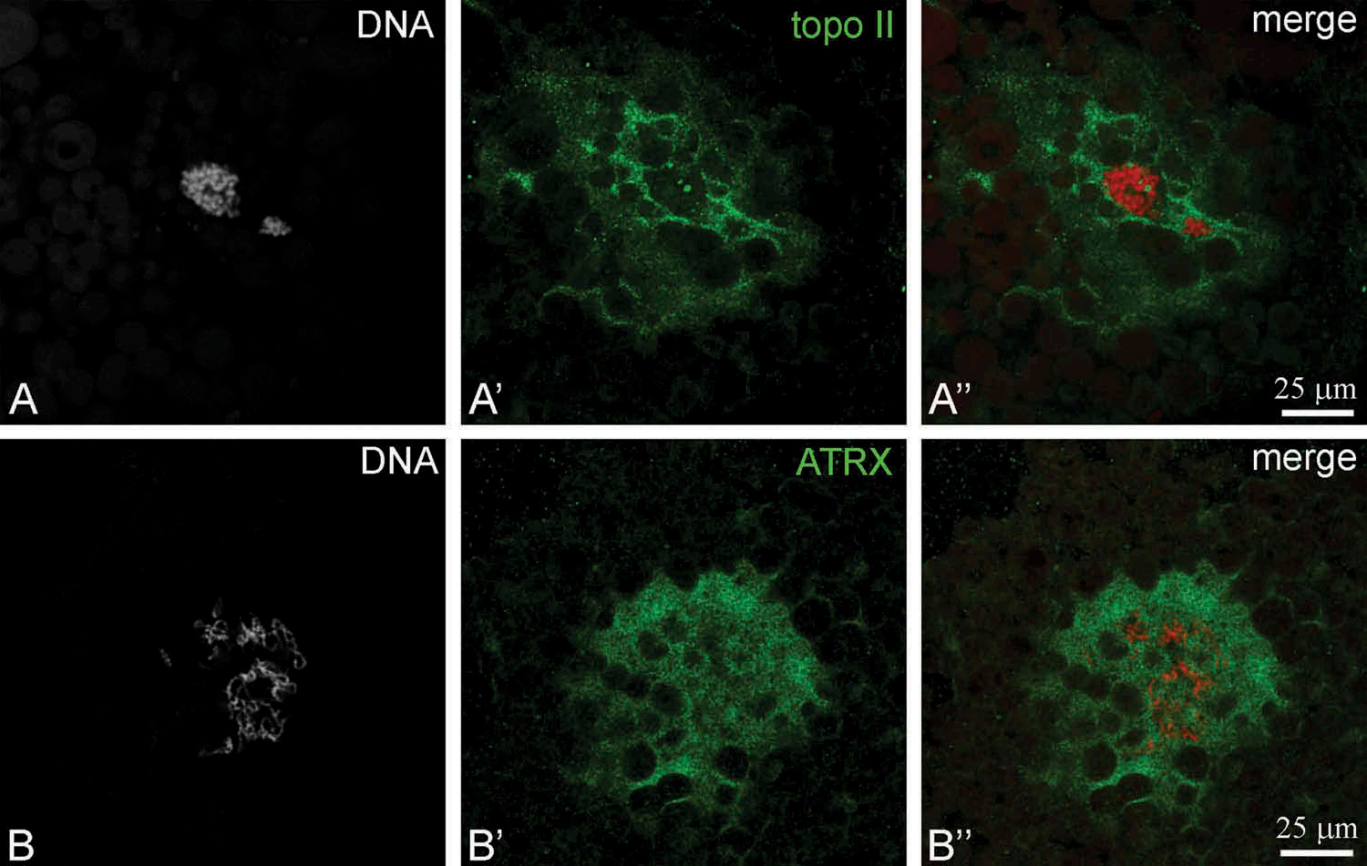
Localization of DNA topoisomerase II (topo II) (a) and ATRX (b) in the karyosphere capsule of *Rana temporaria* oocytes. DNA (karyosphere) is counterstained with DAPI (*red*).

Since the formation of the karyosphere during the long diplotene stage of meiotic prophase includes deep topological chromatin transformations, we also studied the localization of topoisomerase II (topo II), one of the key enzymes necessary for the segregation of chromosomes in meiosis [29]. The antibody against topo II stained the KC rather than the karyosphere itself (Figure 4(a)). The KC was also stained brightly with an antibody against ATRX, an essential protein involved in chromatin remodeling (Figure 4(b)). Only scarce small dots of ATRX were observed in association with the periphery of condensed chromatin, i.e. the karyosphere.

**Figure 5.**
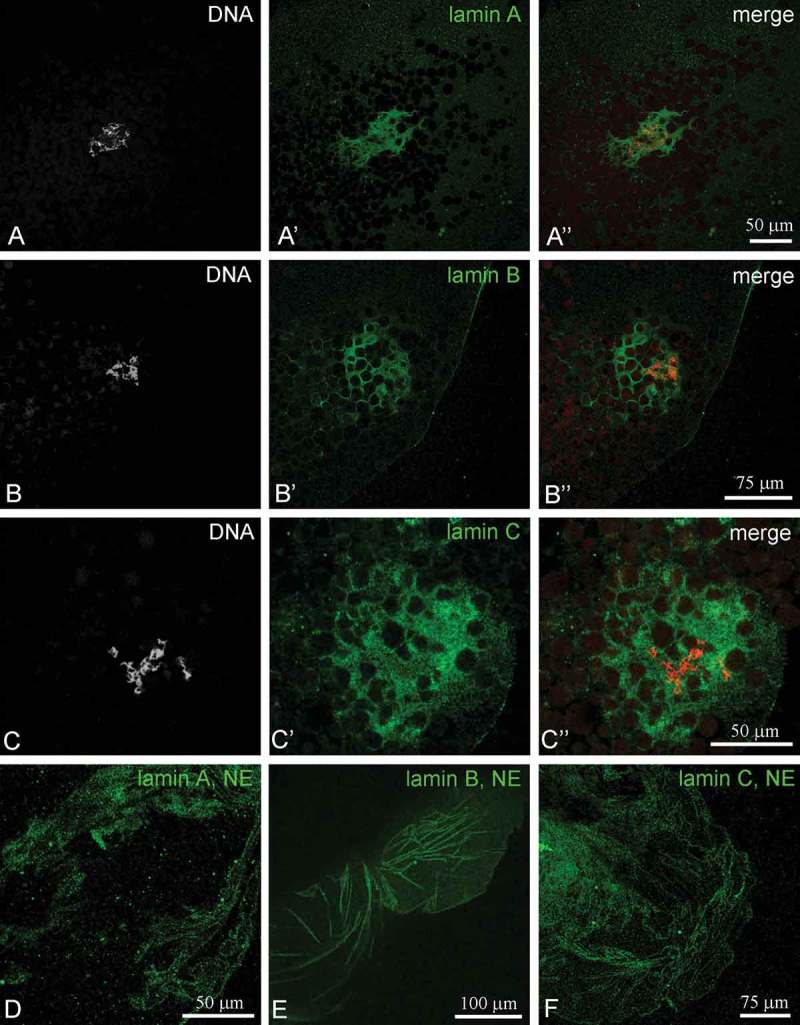
Localization of lamins A, B and C in the nucleus of *Rana temporaria* oocytes. (a-c), karyosphere with capsule; (d-e), fragments of nuclear envelope (NE). DNA (karyosphere) is counterstained with DAPI (*red*).

**Figure 6.**
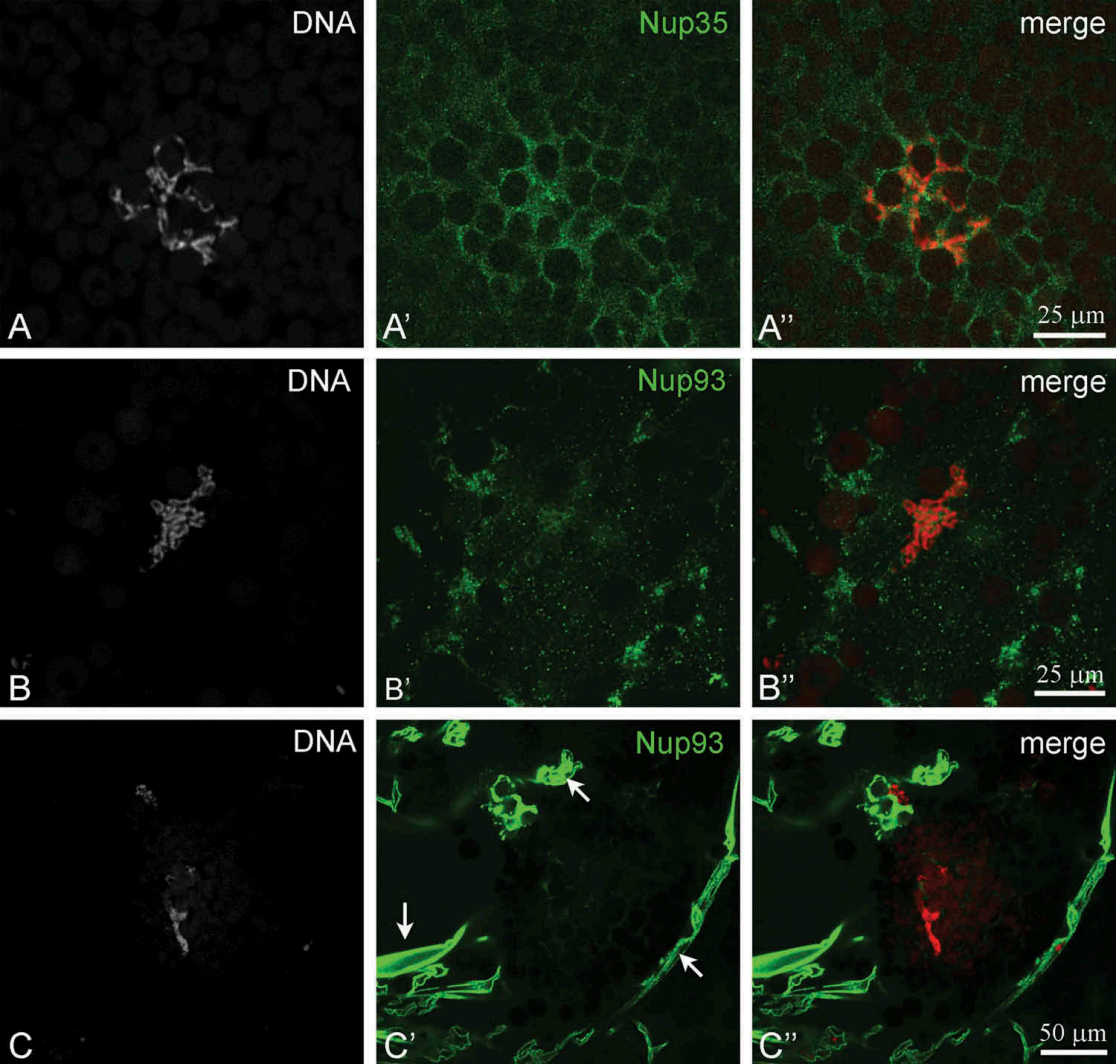
Localization of nucleoporins Nup35 (a) and Nup93 (b,c) in the nucleus of *Rana temporaria* oocytes. DNA (karyosphere) is counterstained with DAPI (*red*). In C, remnants of nuclear envelope (*arrows*) are seen in the same preparation.

Morphological studies [12,13] have provided evidence that the KC in *R. temporaria* oocytes contains some structural counterparts of the NE, including autonomous pore complexes assembled into ‘pseudomembranes’, so we used a set of antibodies against several common proteins associated with the NE. Among the structural proteins, lamins A/C and B are also abundant in the KC (Figure 5(a-c)). The presence of lamins in the KC was confirmed by bright staining of the remnants of the nuclear envelope (NE), often visible in the same preparations; in a conventional manner, all three types of lamins were found in association with the NE (Figure 5(d-f)). Additionally, antibodies against the nucleoporins Nup35 and Nup93 stained the inner part of the nucleus and the remnants of NE independently of the oocyte stage (Figure 6(a-b)). Anti-Nup35 antibody stained a fibrous material in the KC, suggesting the presence of Nup35 in this material, while Nup93 was mostly revealed in membrane fragments (Figure 6(c)). The images of Nup93 staining were very similar to those of lamins A and C.

**Figure 7.**
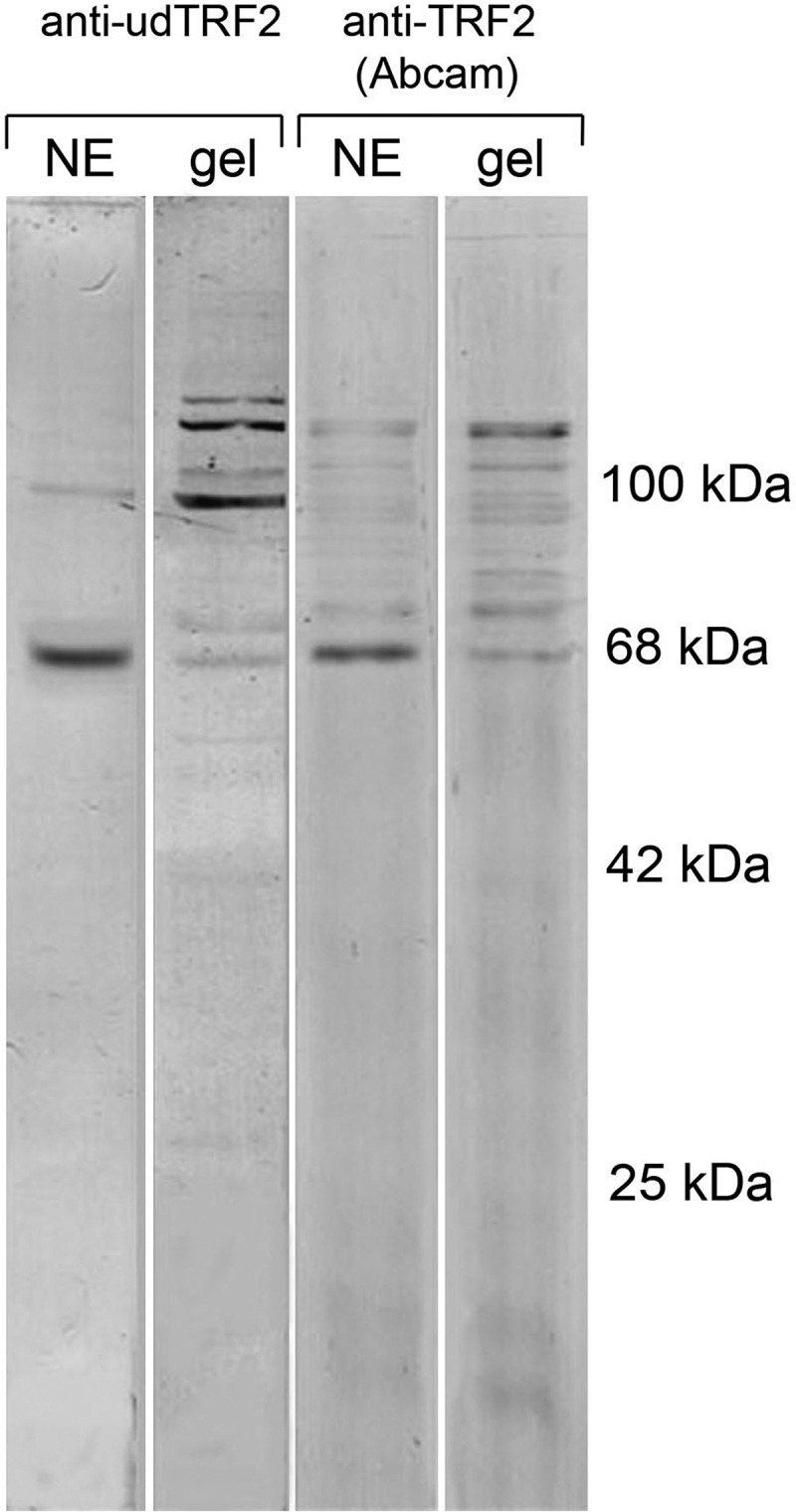
Immunoblotting of the frog oocyte nuclear envelopes (NE) and inner parts of the nuclei (nuclear gels) with anti-udTRF2 antibodies and commercial antibodies to TRF2 (Abcam).

**Figure 8.**
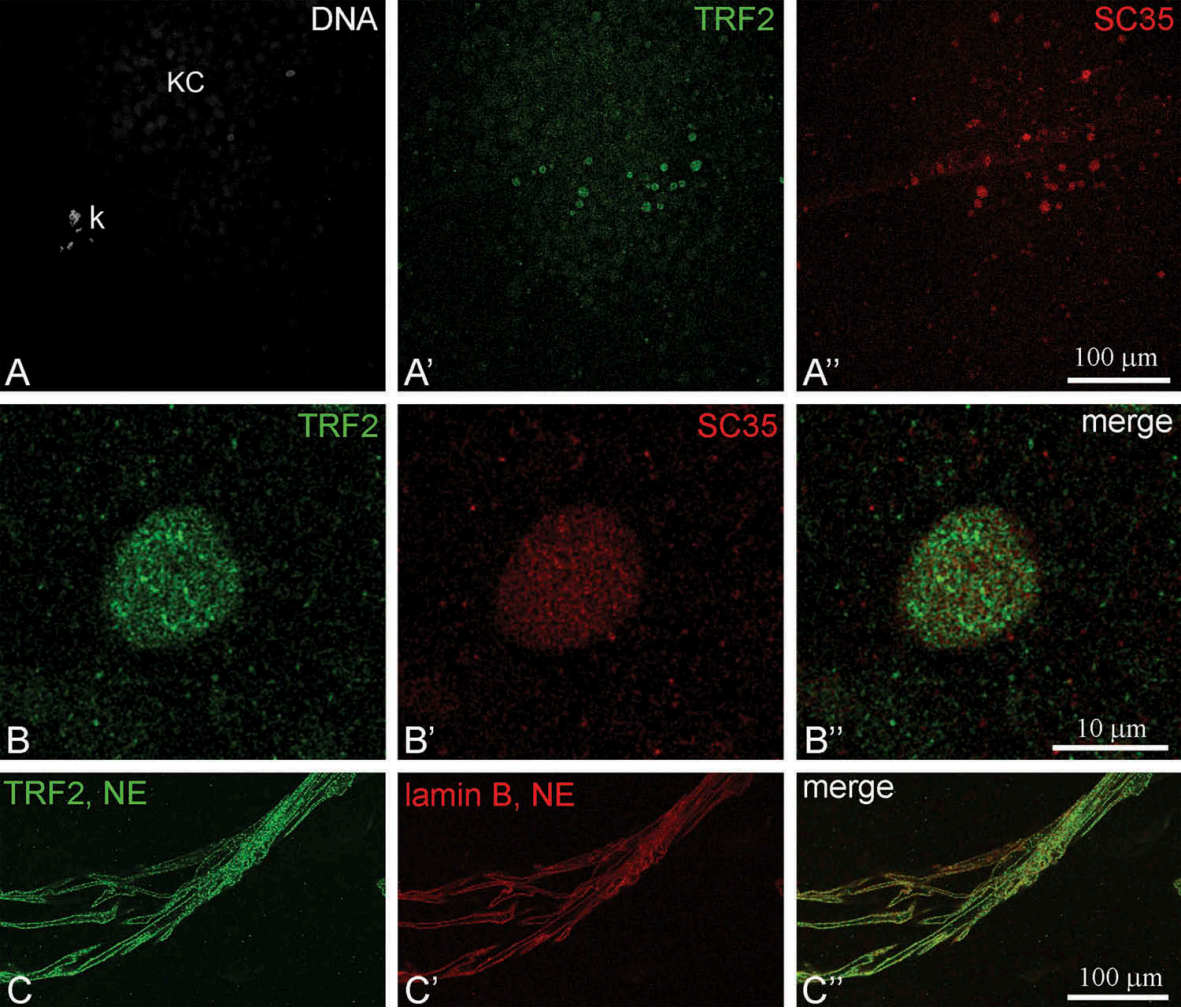
Localization of TRF2 in the oocyte nucleus of *Rana temporaria*. (a), General view on the karyosphere (k) with capsule (KC). TRF2 (*green*) predominantly localizes to SC35-containing bodies (nuclear speckles, *red*) associated with the capsule. DNA is counterstained with DAPI (*gray*). (b), Nuclear speckle at higher magnification to show that TRF2 is not colocalized with SC35. (c), Colocalization of TRF2 (*green*) and lamin B (*red*) in the remnant of the nuclear envelope (NE).

The membrane telomere-binding protein TRF2 was initially isolated from *R. temporaria* late oocytes NE [19], but in mouse oocytes, TRF2 has been found in SC35 domains [30]. In immunoblots, the commercially available antibody recognized some minor zones with an apparent molecular mass higher than TRF2 itself, apparently an unprocessed form of the protein. The anti-udTRF2 antibody clearly distinguished ordinary TRF2 in the NE and TRF2 in complexes of the high Mw form in the inner part of the oocyte nucleus (Figure 7). With these antibodies, we also found TRF2 in the inner part of the KC. TRF2 was found to localize in most of the SC35-containing domains (speckles) including those associated with the KC, which displayed bright staining (Figure 8(a)). Double staining revealed that there was no co-localization of TRF2 with SC35 inside the speckles (IGCs, SC35 domains, Figure 8(b)), while in the NE, TRF2 co-localized with lamins (Figure 8(c)).

## Discussion

### Karyosphere and transcription activity

In grass frog late oocytes, condensed chromosomes that are assembled into a karyosphere and embedded into a KC demonstrate a high level of the histone modification H3K9me3, a key mark of transcriptionally repressed chromatin [31]. However, the chromosomes in stage 5 oocytes still incorporated BrUTP, demonstrating residual transcription. However, BrUTP incorporation was undetectable in stage 6 oocytes, which suggests that the karyosphere becomes transcriptionally silent in oocytes finishing their period of growth. This finding is in agreement with [^3^H]-uridine autoradiography data obtained earlier [32], indicating that oocyte chromosomes in the frog are still capable of RNA synthesis at the beginning of karyosphere formation, but their transcriptional activity is reduced by approximately three-fold and almost entirely ceases in the winter season when the karyosphere terminates its development.

The incomplete cessation of transcription generally characterizes karyospheres with a capsule, such as in the oocytes of the lacewing *Chrysopa* [33] and the red flour beetle *Tribolium* [17]. Conversely, karyospheres without a capsule (karyosomes) become transcriptionally inert at an early stage of development [34]. Known exception includes *Drosophila* oogenesis, in which the karyosome resumes RNA synthesis for a brief period in stage 9–10 oocytes and then becomes compact and transcriptionally inert again [35,36]. It cannot be excluded that the KC could be a specific attribute of the oocyte nucleus when residual transcription is maintained for a prolonged period at the diplotene stage. In the case of early inactivation of the chromosome apparatus at the beginning of the diplotene stage, different nuclear bodies usually play the role of storage compartments for inactive factors disengaged from gene expression; this has been proposed for insect oocytes [37]. As a rule, the KC is absent in this case. If the karyosphere retains residual transcription for a long period of the diplotene stage, the KC along with nuclear bodies such as nuclear speckles can serve as an additional storage compartment for such molecules.

It remains unknown as to which DNA sequences are transcribed and which RNAs are synthesized when the karyosphere retains residual transcription. It is likely that specific regulatory RNAs required for early embryogenesis of the concrete species are synthesized in this period and accumulate in the KC.

### Karyosphere capsule and nuclear actin

In the present work, we were able to reveal a significant accumulation of actin in the KC of *R. temporaria* late oocytes. We used a set of antibodies against different epitopes of the actin molecule. All three antibodies used in this study stained the KC, although the antibody against the C-terminus also revealed foci associated with condensed chromatin in stage 5. In several papers, different anti-actin antibodies have been shown to detect nuclear and cytoplasmic actin with variable efficiency [38,39]. In particular, the antibody against the C-terminus was more effective at revealing nuclear actin, especially in areas of condensed chromatin in the nucleus of mouse embryos [40]. However, the authors demonstrated that the pattern of actin association with transcription sites does not depend on the type of anti-actin antibody used. They speculated that differences in staining with antibodies raised against the C- and N-termini of actin molecules are not due to selective labeling of functionally different actin forms, as previously believed [41], but could be linked to the physicochemical properties of the antibodies.

We observed significant alterations in actin staining after treatment with CytD, which interferes with the dynamics of actin polymerization by binding to the barbed end of actin filaments [42]. The C-terminus of actin is closer to the barbed end than the N-terminus [43]. So, CytD and antibodies against the C-terminus compete for binding to the barbed end, so the actin molecule is not available for antibody binding.

CytD treatment also showed that a significant portion of actin in the KC is in the F-form, since anti-actin antibodies stained only shreds of the KC after CytD treatment. F-actin is essential for stabilizing the mechanical integrity of *Xenopus* oocyte nuclei [44,45]. The presence of actin bundles in the nucleus of *R. temporaria* late oocytes has been documented by different methods including immunoelectron microscopy, but little attention has been paid to the karyosphere [46–48]. A high concentration of F-actin in the *R. temporaria* KC was clearly visible after phalloidin staining (Figure 3). Apart from the frog, F-actin was revealed as a key structural protein of the KC in the oocytes of some insects, including six species of green lacewings [49], the apple blossom weevil [50] and the red flour beetle [17]. Thus, F-actin may be considered as a signature component of the KC, which, in turn, is an evolutionarily conserved structure of the oocyte nucleus.

### Karyosphere capsule and DNA topoisomerase II and ATRX

In late *R. temporaria* oocytes, we detected a high concentration of topo II and ATRX in the KC. Since the KC *a priori* contains no DNA, the finding of DNA-associated proteins in the *R. temporaria* KC is intriguing.

Topo II is a key enzyme necessary to resolve topological problems that arise when the chromatin structure changes during DNA replication and transcription and the segregation of mitotic and meiotic chromosomes. It can relax supercoiled DNA in an ATP- and Mg^2+^-dependent manner and represents one of the first proteins identified in the scaffold of mitotic chromosomes [51,52], demonstrating highly dynamic behavior during mitosis [53]. In meiotic cells, in particular, in diplotene oocytes of mice, topo II is abundant in the nucleoplasm, but absent in the central body of the mouse karyosphere [30]. In birds, topo II is concentrated in protein bodies associated with the centromere regions of lampbrush chromosomes and, in the late diplotene, in the central body of the bird karyosphere [54]. Unlike the mouse, the central body of the karyosphere of birds arises due to the fusion of these specific centromere protein bodies following the lampbrush stage. In both cases, no KC exists. Topo II is definitely a component of the KC in *R. temporaria* late oocytes. However, a high concentration of topo II in both the protein bodies of birds and the KC of *R. temporaria* does not evidently reflect its involvement in chromatin condensation, as these extrachromosomal entities associated with the karyospheres of different types may be storage sites to regulate the topo II concentration in the oocyte nucleus.

Another conspicuous component of the *R. temporaria* KC was found to be alpha-thalassemia/mental retardation X-linked protein (ATRX). This essential ATPase/DNA helicase, a member of SWI/SNF family, is one of the key chromatin remodelers [55]. A role of ATRX in the processes of karyosphere formation and maintenance was established for mammalian oocytes [56] in which the patterns of ATRX distribution reflected the changes in the transcriptional activity of chromatin during its compaction into the karyosphere. ATRX has been shown to determine the cellular mechanisms activated in response to chromosome instability and is required for silencing of major satellite transcripts in the maternal genome [57]. Loss of maternal ATRX results in chromosome instability in mouse oocytes and early embryos [58]. In mouse embryogenesis, the dynamics of ATRX reflect the processes of nuclear reorganization related to the gradual activation of chromosomal transcription [59,60]. Genome inactivation in the final steps of *R. temporaria* karyosphere development can result in the accumulation of ATRX in extrachromosomal elements such as the KC. This is an evolutionarily conserved feature of oogenesis, since the accumulation of ATRX in the KC and/or in the nuclear bodies of certain types has been documented in the late diplotene oocytes of some insects, regardless of the structure of their karyospheres [61].

### Karyosphere capsule and proteins associated with the nuclear envelope

Nuclear pore complexes are composed of multiple copies of ~ 30 different nucleoporins that display both ubiquitous and cell type-specific functions during development. In vertebrates, Nup35, also known as Nup53, was previously described to interact with Nup93, Nup155 and Nup205 and is required for NE assembly *in vitro* [62]. We were able to reveal Nup35 and Nup93 in both the KC and in the fragments of the NE visible in the same preparation, suggesting that some nucleoporins are indeed components of the KC. This seems to confirm the analogy of the ‘pseudomembrane annuli’ of the KC and the pore complexes of the NE in the nucleus of *R. temporaria* oocytes.

We also found that lamins A/C and B, the proteins of intermediate filaments that underlay the NE to form the nuclear lamina, represent other abundant structural proteins of the *R. temporaria* KC. In the frog, lamins of all three types were found to be present in both the KC and the NE. Since the presence of lamin B was previously documented for certain ultrastructural elements of the KC in beetle oocytes [17], lamins can be considered as evolutionarily conserved constituents of the KC.

In mammals, there are cells without lamin A, but no cells without lamin B exist. T-cells and B-cells express only lamins of type B. Undifferentiated human and mouse embryonic stem cells lack lamins A/C, but express lamins B1 and B2 [63]. B-type lamin is expressed throughout embryogenesis, whereas lamin A and lamin C are not expressed until the tissue differentiation stage of development [64]. In the nucleus of mouse oocytes derived from antral follicles, a significant portion of lamin A is stored in a nucleolus-like body, i.e. the central body of the karyosphere [30]. It is likely that the material stored in different extrachromosomal structures associated with the karyosphere, including the KC in the frog and the central body in the mouse, may be used to organize chromatin before fertilization.

We localized telomere-binding factor 2 (TRF2) using an antibody produced against the TRF2-specific domain udTRF2 [26,27]. This antibody was used to reveal a high Mw, probably unprocessed form of TRF2. Anti-udTRF2 immunostaining was much more intensive in SC35-containing nuclear bodies (splicing speckles) rather than the KC, suggesting that SC35 domains are the sites for TRF2 accumulation in the nucleus of *R. temporaria* late oocytes. Similarly, TRF2 also dominates in the nuclear speckles of mouse oocytes until the end of diplotene, when a portion of TRF2 begins to accumulate in the central body of the karyosphere [30]. Thus, the same extrachromosomal structures function as storage compartments for TRF2 independently of the karyosphere type.

## Conclusion

The present study extends the concept of the KC as a unique superstructural formation of the nucleus of late oocytes in both vertebrates and invertebrates. The KC does not develop in all cases of karyosphere formation, and the molecular composition and biological significance of this peculiar structure of the oocyte nucleus remain elusive. The KC is indeed a specialized component of the oocyte nucleoskeleton that possesses some essential structural proteins such as F-actin and lamins. Nuclear pore complex proteins such as Nup93 and Nup35 are also detectable in the KC and could be constituents of ‘pseudomembranes’. At the same time, the KC is the storage place for the DNA-related proteins, such as topo II and ATRX, which are probably required for chromatin organization during further meiotic divisions. We found that nuclear speckles (IGCs, SC35 domains) represent a storage place for TRF2 in frog oocytes (with a KC) as well as in mouse oocytes (with a central body of the karyosphere) [30]. The KC may contain many other multifarious proteins and RNAs, but a complete list of these as well as the roles of these molecules in further meiotic events and/or early embryogenesis are yet to be established.
